# Effect of silylating agents on the superhydrophobic and self-cleaning properties of siloxane/polydimethylsiloxane nanocomposite coatings on cellulosic fabric filters for oil–water separation†

**DOI:** 10.1039/d0ra10565a

**Published:** 2021-03-04

**Authors:** Ubong Eduok, Omar Faye, Jerzy Szpunar, Mazen Khaled

**Affiliations:** Department of Mechanical Engineering, College of Engineering, University of Saskatchewan 57 Campus Drive Saskatoon S7N 5A9 Saskatchewan Canada ubong.eduok@usask.ca +1 306 966 5427 +1 306 966 7752; Department of Chemistry and Earth Sciences, College of Arts and Sciences, Qatar University P.O. Box 2713 Doha Qatar

## Abstract

A new facile approach for preparing two nonfluorinated hybrid organic–inorganic siloxane/polydimethylsiloxane nanocomposite coatings for cotton fabrics is presented using two distinct silylating agents. The first coated fabric was prepared predominantly *via* trimethylsilyl modification using hexamethyldisilazane (HMDS) while higher amounts of trimethoxy(octadecyl)silane (TMOS) further enhanced the superhydrophobicity of the second coating matrix. Unlike HMDS with substituted silyl (Me_3_Si) groups, TMOS consists of hydrolysable trimethoxy silyl ((MeO)_3_Si) chemical groups that allowed for the formation of nanosilica with Si–O–Si linkages needed to foster stable coatings. After characterization and testing, these coated fabrics demonstrated varying responses to harsh solvents and thermal conditions. Both sets of coated fabrics exhibited unique capacities for self-cleaning and oil–water separation as superhydrophobic filters due to (a) their low surface energy silylated hybrid polysiloxane chemical groups, (b) their highly reduced surface wettability and (c) nanopatterned surface morphologies. In this study, coated superhydrophobic cotton fabrics revealed a higher static aqueous contact angle 
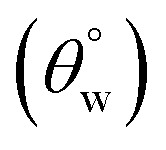
 of more than 150° and sliding hysteresis angle 
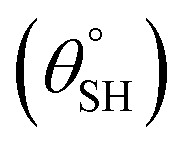
 of less than 5°. Coated fabrics with 30 mg TMOS/10 mg HMDS (CMF3) and 30 mg HMDS/10 mg TMOS (CTF3) exhibited optimal superhydrophobicity. Both fabrics also retained percentage separation efficiencies over 90% for both chloroform–water and toluene–water mixtures. However, CTF3 displayed 
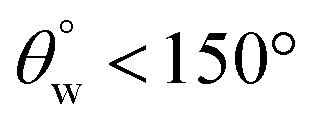
 with a recorded separation efficiency less than 90° after five filtration cycles.

## Abstract 2

Two silylating agents, hexamethyldisilazane and trimethoxy(octadecyl)silane enhance the superhydrophobicity and self-cleaning properties of siloxane/PDMS nanocomposite coatings on cotton filters for oil–water separation.

## Graphical abstract

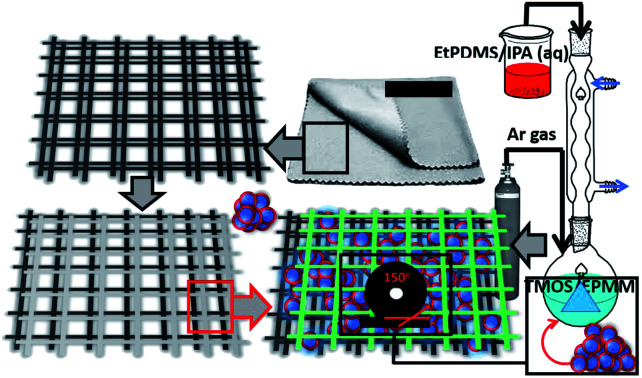

## 1. Introduction

Recent trends in human activities have led to frequent oil spills and waste disposals, and these have severely impacted on marine lives.^1^ Discharges from industrial wastewaters generate oil-based environmental eluents (*e.g.*, toluene) and water-soluble pollutants (*e.g.*, dyes) whose dispersion within the food chain are also detrimental to human health.^2^ In cases of oil spills, the formation of organic films isolates the seawater from rapid oxygen exchange with the surrounding atmosphere while starving marine lives of the needed molecular oxygen sustenance. The challenges associated with effluent oil treatments using physical and chemical techniques as well as the complex nature of these effluents necessitate the design and development of functionalized filter materials capable of efficient and sustainable oil/water separation to remediate severe pollution incidents. The use of superhydrophobic filters has attracted special attention due to their unique surface properties, including low surface energies and self-cleaning capacity toward high separation efficiency between oil and water. Unlike most chemical treatment techniques, superhydrophobic filters utilized in physical separation techniques are environmentally friendly materials that are effective for liquid–liquid phase separations without the need for the use of specialized equipment. These materials reserve their unique recovery efficiencies for oil remnants and high oil–water separation capacity due to their nanostructured superhydrophobic surfaces with high hydrostatic aqueous contact 
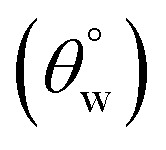
 and low sliding hysteresis 
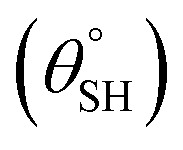
 angles. Modern superhydrophobic filters are fabricated to achieve surfaces with exceptionally low surface energies capable of mimicking the self-cleaning lotus effect in *Nelumbo nucifera* (lotus) leaves, with minimized water and even oil droplet adhesions.^3^ Like surfaces of the lotus leaf, these superhydrophobic filters are designed with non-wetting micro/nanohierarchical papilla-leavened structures with superhydrophobic surfaces.^4^ These superhydrophobic filter surfaces are fabricated from various techniques involving physical changes, chemical depositions, spray, vapor and even laser surface modifications.^5–8^ Functional superhydrophobic filter surfaces fabricated from these techniques are also designed to possess defined textures with chemical properties capable of inducing extremely high-water repellency to which cotton fabrics are a classical example. Cotton fabrics are one of the most low-cost and efficient superhydrophobic filters since they consist of hydrophilic hydroxyl-bound cellulosic backbones capable of being chemically altered to introduce new functional groups that offer enhanced surface properties.^9^ They are also flexible and with enhanced tunable mechanical strengths.

There are a number of recent research reports and review articles featuring oil/water separation and self-cleaning properties of cellulosic superhydrophobic fabric materials.^10–17^ Guo *et al.*^14^ investigated the efficiency of a superhydrophobic bilayer coating deposited on cotton fabric *via* spraying technique. The coating layers consisted of TiO_2_/PDMS and alkylammonium functional silsesquioxane/phytic acid hybrid composites. Results reported by authors revealed a coating system with significant capacity for self-cleaning and water–oil separation due to its superhydrophobic surface. The coating system with the best performance was abrasion resistant and also exhibited strong separation efficiency for strong acid– or alkali–oil mixtures. This last function stood at 
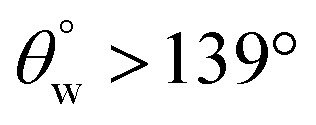
 after 50 abrasion cycles due to PDMS modification of the adhering TiO_2_ nanoparticles. Jannatun *et al.*^5^ have also reported the use of a PDMS-based coating system that utilizes boric acid (H_3_BO_3_) as a cross-linker between silica nanoparticles and poly(vinyl alcohol). It was applied on the cotton fabric *via* dip coating technique. The presence of SiO_2_ nanoparticles within the coating also contributed to its oil–water separation and self-healing abilities. Variants of this coated fabrics revealed durability against mechanical abrasion (*e.g.*, tape-peeling) and chemical stresses (*e.g.*, corrosion solvents) with sufficiently unaltered values of 
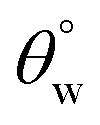
 after several cycles. A magnitude of separation efficiency up to 90% was recorded during oil–water mixture separation between 5 to 45 separation cycles. In another study, Talebizadehsardari *et al.*^13^ investigated the efficiency of reusable oil/water separation when using a superhydrophobic nanocomposite coated cotton fabric fabricated from PDMS and silica nanoparticles. Here, authors also probed the effect of curing agents on the oil/water separation efficiencies of coated fabrics. They observed that curing agents contributed to the superhydrophobicity of each coating variants as more silica nanoparticles were adsorbed on the fibers of the fabric. Authors reported 97.6 and 95.1% separation efficiencies after 5 and 10 washing cycles, respectively.

One common highlight among these reported examples is the introduction of nanoparticles to promote superhydrophobicity. However, there is a pressing concern regarding failed coating adhesion when their particle sizes and concentrations are not sufficiently regulated. In the present study, steps are taken to further to achieve superhydrophobicity by addressing this inherent surface problem by modifying coated polymer chains with silylating agents. As a contribution to the use of reinforced coated superhydrophobic fabrics, the present study features a facile one-pot novel approach to synthesizing new siloxane/PDMS nanocomposite coatings involving surface modification with two distinct silylating agents to achieve enhanced superhydrophobicity when deposited on cotton fabric. The two silylating agents are hexamethyldisilazane (HMDS) and trimethoxy(octadecyl)silane (TMOS). Unlike HMDS with substituted silyl (Me_3_Si) groups, TMOS consists of hydrolysable trimethoxy silyl ((MeO)_3_Si) chemical groups that allow for the formation of nanosilica with Si–O–Si linkages needed to foster stable coatings. Why use silylating agents? These precursors were introduced as silicon-based components capable of improving the surface hydrophobicity of the bulk coating *via* their substituted silyl group (R_3_Si) chemical groups. Here, the desired substituted silyl groups are introduced within molecular chains of the synthesized nanocomposite polymer in order to impart physical and chemical modifications to their surfaces. The major research highlight features the investigation of the effects of both silylating agents toward enhanced surface non-wetting, self-cleaning and oil–water separations. The surfaces of coated fabrics in the present study possess functional groups chemically grafted on to the cotton fabrics to offer low wettability and nanoporous and nanopatterned surface roughness.

## 2. Experimental procedures

### 2.1. Reagents and chemicals

The coating precursors in the present study were ethoxy terminated polydimethylsiloxane (EtPDMS, 5–10 cst, >95%) and 3-(2,3-epoxypropoxypropyl)methyldimethoxysilane (EPMM, 95–100%), purchased from GELEST. Trimethoxy(octadecyl)silane (TMOS, >95%), hexamethyldisilazane (HMDS, ≥99%) and chlorotrimethylsilane (TMCS, ≥97%) were purchased from Sigma Aldrich and GELEST, alongside isopropyl alcohol (IPA, HPLC grade), acetone (≥99.5%) and anhydrous ammonia (≥99.98%). Sudan red G (analytical standard) and methyl orange (ACS reagent, 85% dye content) from Sigma Aldrich were utilized as dyes in this study. All the chemicals in the present study were used as purchased without further purification as they were analytical grade regents.

### 2.2. Preparing silylated superhydrophobic silica nanocomposites

**Fig. 1:**
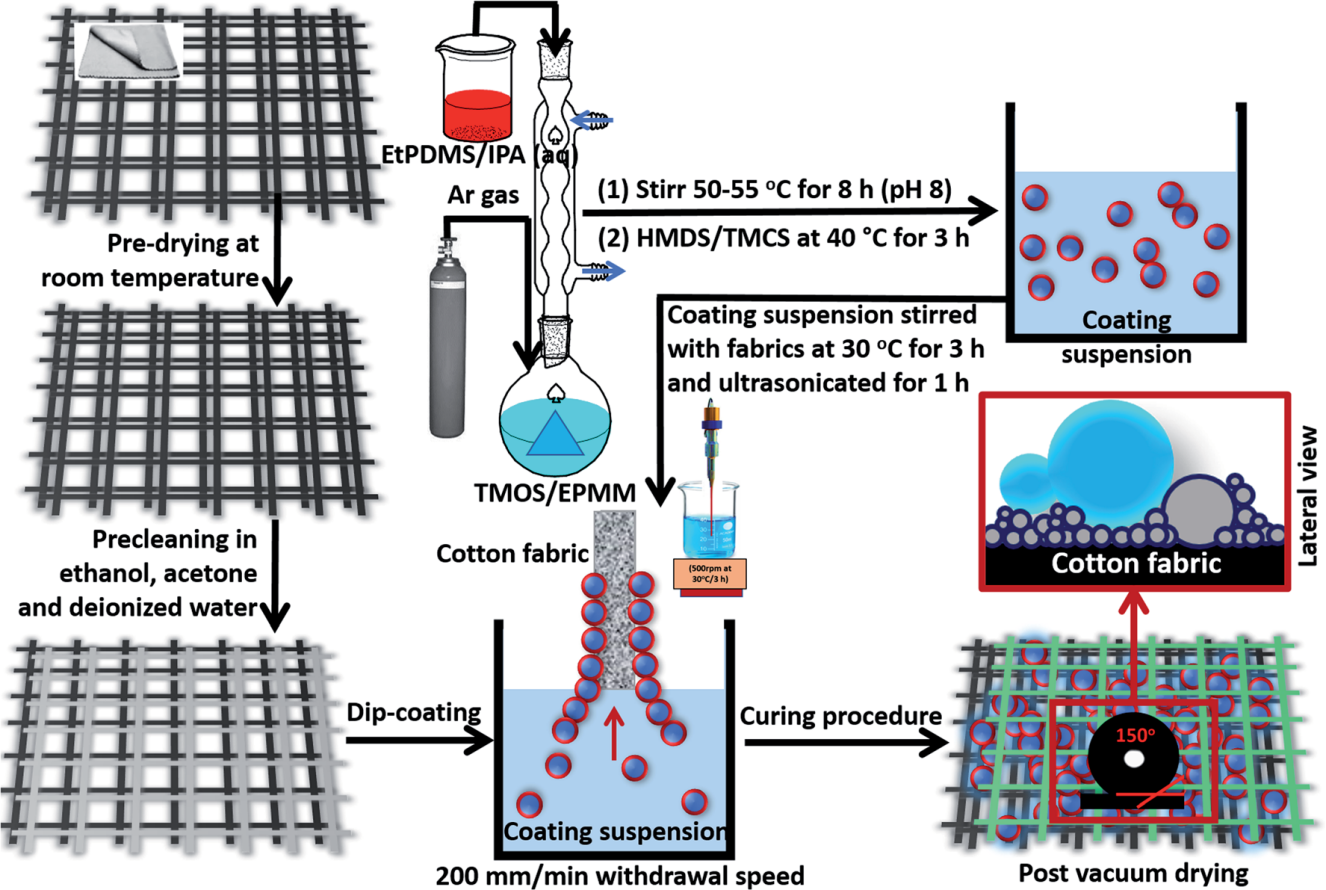
An annotated schematic showing the preparation procedure for silylated superhydrophobic siloxane/PDMS hybrid nanocomposite coatings on cotton fabrics reported in this study. The process starts from the actual synthesis of silylated superhydrophobic coatings, precleaning procedure of all cotton fabric substrates and surface modification of precleaned cotton fabrics by dip-coating technique.

Cotton fabrics were modified with coatings synthesized *in situ* a reaction medium with two different silylating agents, introduced to provide the needed surface hydrophobicity. The first step in preparing the PDMS coating involved refluxing a suspension of trimethoxy(octadecyl)silane (TMOS) and 3-(2,3-epoxypropoxypropyl)methyldimethoxysilane (EPMM, 10 mg), in equimolar concentrations with ethoxy terminated polydimethylsiloxane (10 mg) in 3 mL water and 2 mL IPA at 50–55 °C. The pH of the reaction medium was adjusted to 8 using ammonia solution. After allowing hydrolysis–condensation reactions on respective alkoxide-bearing reactants for 8 h, we proceeded with modification of the reaction medium with hexamethyldisilazane (HMDS) in the presence of chlorotrimethylsilane TMCS (2 : 0.5) at 40 °C for 3 h and stirred at 500 rpm. By this time, the resultant reaction medium turned viscous. Varying concentrations of HMDS and TMOS (10–40 mg) in the reaction media represent in coating variants in the present study. Labels were provided according to the amounts of both silylating agents, HMDS and TMOS, within the modified coatings as presented in Table 1. The synthesis schematics for these silylated superhydrophobic siloxane/PDMS hybrid nanocomposite coatings and their fabrication scheme for fabric surface modification are summarized in Fig. 1.

**Table 1:** Coating labels and their description as utilized in the present study

Coating matrix	Variant labels/notations	Coating description
CTF1–4	CTF1	10 mg HMDS/10 mg TMOS
	CTF2	20 mg HMDS/10 mg TMOS
	CTF3	30 mg HMDS/10 mg TMOS
	CTF4	40 mg HMDS/10 mg TMOS
CMF1–4	CMF1^a^	10 mg TMOS/10 mg HMDS
	CMF2	20 mg TMOS/10 mg HMDS
	CMF3	30 mg TMOS/10 mg HMDS
	CMF4	40 mg TMOS/10 mg HMDS

a Alongside 20 mg EPMM and EtPDMS, each, and double the cuing duration.

### 2.3. Preparing fabric surfaces modified with silylated silica nanocomposite coatings

Prior to fabric surface modifications, clean cotton fabrics (5 × 5 cm) were prewashed with ethanol, acetone and deionised water, separately, through ultrasonication (Branson M1800). This procedure was repeated three times in order to completely remove adhering surface contaminants and those embedded within the cotton fabrics. These fabrics were then dried at 30 °C for 15 min and then stored up for further use. They appeared clean with characteristic almost crispy feel when immediately removed from the vacuum oven, however, their physical forms were unaltered from this thermal treatment process. These superhydrophobic coatings were separately stirred with each fabric completely immersed within them at 30 °C for 3 h and ultrasonicated for 1 h. This ended the surface modification process. As represented in Fig. 1, the coated fabrics were removed at a 200 mm min^−1^ withdrawal speed (KSV NIMA Dip Coater). This coating technique represents a low cost and reliable method for depositing wet coating films by simply immersing the solid fabric substrates within the coating suspension and withdrawing them at constant speed. This was closely followed by surface curing of coated fabrics using vacuum drying (Thermo Scientific Vac Oven 12.5L) at 40 °C for 1 h in order to remove unreacted and excess solvents on the fabric surfaces. They were also labelled accordingly and stored under a desiccator tor for further use.

### 2.4. Characterization of silylated superhydrophobic coated fabric filters

Field emission scanning electron microscopy (Hitachi SU6600 SEM) was utilized in analyzing the surface morphologies of both uncoated and all coated fabrics at appropriate acceleration voltage (10–20 kV). Functional group chemistries of adhering coatings on the fabrics were also probed by means of Fourier Transform Infrared (FTIR) spectroscopy and X-ray photoelectron spectroscopy (XPS). These techniques also accounted for the degree of chemical conversions between reaction precursors and the resulting coating composite products for each synthesis step. FTIR spectroscopic analyses were conducted using a Renishaw Invia Reflex Raman microscope (with FTIR) equipped with an IlluminatIRII FTIR microscope accessory (Smith's Detection, Danbury, CT). Spectral plots were recorded at a 4 cm^−1^ spectral resolution between 400 and 4000 cm^−1^ wavelength range with a system that uses a 36× diamond ATR objective (10–50 μm aperture). The Invia Reflex used was a 514.5 nm laser excitation. X-ray Photoelectron Spectroscopy (XPS) analyses of both uncoated and coated fabrics were conducted using Kratos AXIS Supra XPS; all coated material surfaces were compatible with its ultra-high vacuum (UHV). The instrument is equipped with an Al Kα monochromatic X-ray source. All tests were carried out at a 90 deg on 120 micron spot size. X-ray scans for high resolution measurements proceeded using 0.05 eV steps with pass energy, 15 keV accelerating voltage and 15 mA emission current, respectively. The present study depended on evaluating inherent changes on the coated surfaces after various treatments. Here, the aqueous contact 
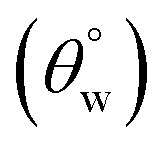
 and sliding hysteresis 
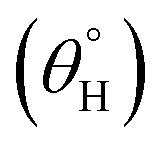
 angles of respective coated fabrics were measured using Data Physics contact-angle meter. This was measured after carefully placing approximately 5 μL tiny deionized water drops on each fabric surface at ambient temperature. The presented results in this study are mean value of five measurements.

### 2.5. Tests for chemical stability and self-cleaning abilities of superhydrophobic cotton fabric filters

These silylated superhydrophobic coated cotton fabrics were completely exposed to 50 mL HCl and NaOH (one molar each) for 24 h in order to test their chemical stabilities. After each test within respective solvents, each fabric was repeatedly rinsed with absolute ethanol, dried in vacuum for 1 h at 35 °C before measuring its surface aqueous contact angles. Similar tests were also conducted after exposure to defined thermal conditions in vacuum between 40 and 70 °C. The self-cleaning ability of the best performing superhydrophobic coated fabric was also examined after placing finely ground powders of colored chalks. This coated fabric was placed on glass slide with equal tilt angle in Petri dishes alongside an uncoated fabric. Water droplets were then carefully introduced from a Diamond™ Jr Mini Pipettor (Globe Scientific) and allowed to roll through these fabric surfaces while monitoring the extent of wetness and powder/dirt accompanied roll-off.

### 2.6. Oil–water separation with superhydrophobic cotton fabric filters

The percentage separation efficiencies of these silylated superhydrophobic coated fabrics were also determined for oil–water mixtures. Here, the coated fabrics were utilized as separation membranes in a filtration test involving repeated cycles after individually fitted appropriately to a laboratory-fabricated filtration set-up. The fabrics were firmly secured between the volumetric glass and the round bottom flask in order to allow the filtrate pass through to the flask without spillage while also retaining the reduce. To simulate a reliable oil–water mixture, chloroform, toluene and water samples were utilized in this test. Chloroform and toluene were separated individually from 50 mL of each oil–water mixture per cycle. Firstly, chloroform acted as the heavy oil phase mixed with an aqueous (*i.e.*, water) phase. This chloroform–water mixture was allowed to pass through the setup and separated accordingly after pouring it through a funnel. Since both liquids are colorless and almost very difficult to observe the separation of both phases within the setup, it was pertinent stain one of them with a dye. By staining water with 5 wt% methyl orange, the aqueous phase possessed a near orange coloration, allowing for a distinct chloroform–water separation toward fostering a good separation efficiency. Methyl orange was soluble only in water and not chloroform, hence, creating a distinct separation boundary between the heavy organic (chloroform) and light aqueous phases. The dyed water remained above membrane due to the superhydrophobicity of the coated fabric filter. However, chloroform filtered through and settled at the bottom of flask by means of gravitational force since it is also heavier than water. This filtration test was conducted for five cycles after rigorous shaking of the chloroform–water mixtures before filtration. This test was also conducted for a dye-stained lighter organic phase (toluene) in combination with water for the same number of cycles and mixture volume (50 mL each cycle). For both toluene/water and chloroform/water mixtures, the separation efficiencies (%) of the superhydrophobic cotton fabrics were computed using eqn (1).^4^1
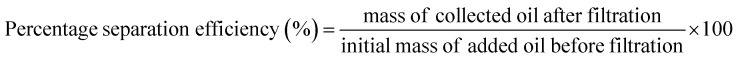


## 3. Results and discussion

### 3.1. Compositional analyses of superhydrophobic coated fabrics

**Fig. 2:**
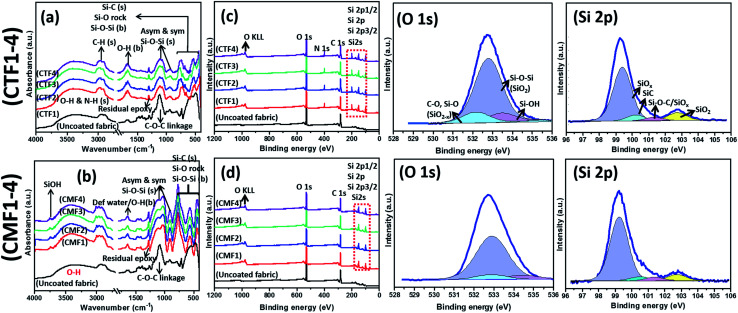
FTIR (a, b) and XPS wide scan (c, d) and deconvoluted high-resolution O 1s and Si 2p curves of coated (CTF1 and CMF1–4) superhydrophobic fabrics.

Compositional analyses of bare and respective coated superhydrophobic fabrics were conducted using FTIR and XPS techniques. The FTIR spectra of CTF1–4 and CMF1–4 coated fabrics are depicted in Fig. 2(a and b). There is a strong adsorption band at 1030 cm^−1^ consistent with symmetric and asymmetric glycosidic linkage stretching COC vibrations and few other cellulose functional groups overlap (*e.g.*, C–C and C–O) (see the FTIR spectrum of the uncoated fabric). There are also broad stretching bands (H connected) from an O–H vicinal between 3000–3600 cm^−1^ corresponding to silanol *n*(Si–OH) groups.^18^ Both peaks are also present on the spectra of coated fabrics with terminal silanols and C–H stretching (from Si–CH_*x*_ alkyl groups) vibrations observed at 3750 and 2976 cm^−1^ shoulders for CMF1–4 compared to CTF1–4. More conspicuous on the spectra of coated fabrics are symmetric and asymmetric Si–O–Si stretching vibrations at 877 and 1044/1086 cm^−1^, respectively. These peaks could be attributed to the formation of polysiloxane network after sol–gel induced condensation reactions from siloxane precursors.^19,20^ There are Si–O–Si bending stretching and Si–O rocking vibrations between 400 and 430 cm^−1^ for both sets of coated fabrics. The presence of Si–C stretching peaks at 1400 cm^−1^ could be attributed to long octadecyl and methyl moieties on coating matrices for CMF1–4 and CTF1–4, respectively.^19,21^ It is the presence of these unreactive organic alkyl groups that contributes to the superhydrophobicity of these siloxane/PDMS silicon hybrid elastomer coatings on the fabrics. Adhering –CH groups on the coated fabrics significantly contribute to their low wetting capacity due to the lowered surface-energy PDMS additive after coating modification.^4^ There are also residual ring-breathing vibration peaks of epoxy at 1255–1260 cm^−1^.

XPS wide scan spectra of respective superhydrophobic coated fabrics are also depicted in Fig. 2(c and d) relative to the uncoated substrate. While there are few peaks consistent with C and O atoms at binding energies of 286.5, 534.4 and 984.6 eV, respectively, there are also core level peaks consistent with the major Si coating components at 97.8 and 150.0 eV.^22^ There are no Si XPS peaks on the bare fabric while the only distinct difference between CMF1–4 and CTF1–4 is in the N peak at 400 eV. Deconvoluted high-resolution spectra for O 1s and Si 2p from coated (CTF1 and CMF1–4) superhydrophobic fabrics are also presented on Fig. 2. The following bonds are identified on the deconvoluted O spectra as O 1s peaks: Si–O–Si from SiO_2_ (533.1 eV), Si–O (531.9 eV) and Si–OH (534 eV).^23^ The observed peaks near 523 eV are related to O atom vacancies at the Si surface^20,21^ due to changes in mobility of the SiO_2−*x*_. Meškinis *et al.*^23^ also pointed out that these peaks may also be linked with SiOC/SiO_2_/SiO_*x*_ and silanol (Si–OH) at approximately, 95% and 5%, respectively. The wide spectra show lower pass energy consistent with fused Si 2p peaks (2p_1/2_ and 2p_3/2_) at 100.8 eV (and Si 2s at 152.86 eV on the wide scan spectra) due to the amorphous siloxane/PDMS phase on both coatings.^24^ There are four designated peaks corresponding to SiO_*x*_, SiC, Si–O–C, and SiO_2_ bonds on the Si 2p spectra on both coatings between 99.6 and 103.5 eV.^23,25^ The SiC peak is an overlap between Si–C and Si–O_*x*_ bonds. Both XPS Si 2p and O 1s spectra reveal the presence of SiO_2−*x*_ and Si–OH states on both coatings, which is also confirmed by FTIR spectra. These XPS results confirms the presence of polysiloxane chemical groups within the surface nanostructures of the fabrics.

### 3.2. Surface morphology of silylated superhydrophobic coated fabrics

**Fig. 3:**
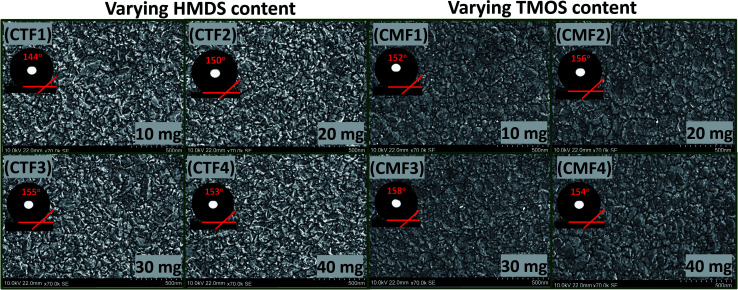
High-magnification FESEM micrographs of superhydrophobic coated cotton fabrics with varying HMDS and TMOS contents.

**Fig. 4:**
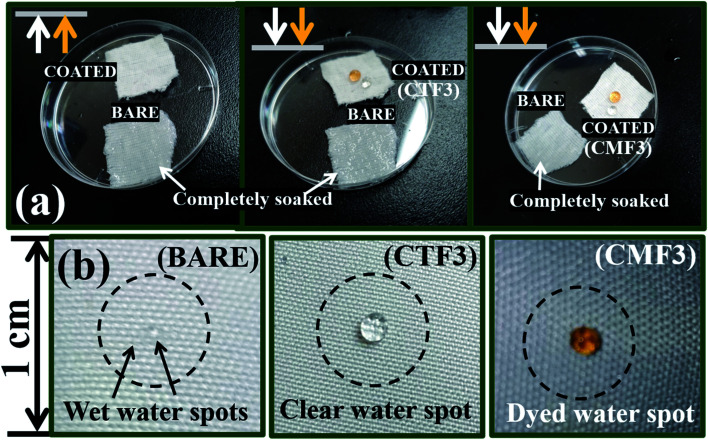
Normal (a) and magnified (b) photographic images showing the differences in surface wettability between bare and superhydrophobic coated cotton fabrics when placed on pure and dyed water droplets (arrows up) and *vice versa* (arrows down). The colour of cotton fabrics in this study is off-white; the observed appearances could be due to varying light shades in the laboratory.

The low- and high-magnification SEM images (from left to right) showing the surface morphologies of bare and coated superhydrophobic fabrics are depicted within the ESI (Fig. S1).† The uncoated fabric displayed a flat morphology with no distinct microfeature. However, there were significant changes in this rather smooth fabric surface after modification with superhydrophobic coatings as displayed on high magnification. These nanocomposites appear to be evenly distributed on individual fabric fibers. SEM image of the coated fabric also reveals a unique morphology with evenly sparse but with rather rough nanoporous structures. It is also worthy of note to mention that variants of this superhydrophobic coating were further prepared according to varying concentrations of the PDMS (10–40 mg). In this study, all coated cotton fabrics were labelled CTF1–4 (10–40 mg HMDS/10 mg TMOS) and CMF1–4 (10–40 mg TMOS/10 mg HMDS) according to the HMDS and TMOS contents. Fig. 3 shows the SEM micrographs of coated superhydrophobic fabric at higher magnifications. The surfaces of these coated fabrics possess numerous porous nanostructures with protrusive surface architectures responsible for trapping air within them, thereby creating a hydrophobic resistance against water penetration. These nanopatterned surface grooves further reduced gross surface energy as the coating morphology contributes to the formation of more air nanobubbles traps within the coatings.^4^ Compared to the bare fabric, the coated fibers showed significant porous nanostructures while their morphologies show distinct nano-hierarchical roughness that also contributes to superior static contact angles. This present study reveals a one-pot approach technique for fabricating a nonfluorinated hybrid organic–inorganic silica nanocomposite coated morphology synthesized after trimethylsilyl modification with hexamethyldisilazane (HMDS) and trimethoxy(octadecyl)silane (TMOS) silylating agents. The presence of these silylating agents promoted enhanced superhydrophobicity when deposited on cotton fabric. It is also worthy of note to mention that coated fabrics were heavier compared to the bare fabric substrate. The differences between CTF1–4 and CMF1–4 could be due to their respective silica contents. The measured weights of all cotton fabrics before any treatment are presented in Table 2. In both coating systems, higher fabric weights were observed with increasing silylating agents (10–40 mg HMDS and TMOS). Preliminary study revealed unique superhydrophobic properties for both sets of coatings. Fig. 4 shows the differences in surface wettability between bare and superhydrophobic coated cotton fabrics when they were placed on pure and dyed water droplets and *vice versa*.

**Table 2:** Individual weights of cotton fabrics substrates (5 × 5 cm) before any treatment

Cotton fabric	Bare fabric	CTF1	CTF2	CTF3	CTF4	CMF1	CMF2	CMF3	CMF4
Weight (g)	10.5	14.1	19.8	21.2	22.5	14.2	20.8	22.8	25.7

### 3.3. Examining the effects of silylating coating contents on the wetting behaviors of coated fabric filters

The ability of these coated surfaces to self-clean depends on their hydrophobicity. Specifically, the contact angle of water on surfaces of this kind represents an important characteristic that also determines the capacity of a coated surface to self-clean. Contact angle is influenced by the microstructural surface roughness, and the Young, Wenzel and Cassie–Baxter's models (eqn (2)) have been developed to describe inherent wettability of self-cleaning surfaces. While the Young's model describes the droplet–surface relations on perfect surfaces, the other two models account for interface effects from rough surfaces. Specifically, the Cassie–Baxter's model of wetting recounts for changes at the interface when adhering water droplets creates air pockets at these surfaces.

2 cos(*θ*_o_) = (*γ*_SA_ − *γ*_SL_/*γ*_LA_); cos(*θ*) = *R*_f_ cos(*θ*_o_); cos(*θ*_CB_) = *R*_f_ cos(*θ*_o_) − *f*_LA_(*R*_f_ cos(*θ*_o_) + 1)

Before examining the effects of silylating coating contents on the collective wetting behaviours of these coated fabric systems, a preliminary water-droplet test was conducted by placing (i) multiple drops of water on exposed coated fabric surfaces and (ii) also by placing these coated surfaces on water spills. The extent of water adsorption is indicative to the hydrophobicity of these fabrics (see Fig. 4(a)). Corresponding photographic images showing the differences in surface wettability between bare and superhydrophobic coated cotton fabrics are also depicted in Fig. 4(b), for both clear and dyed water droplets. These water droplets sat on the coated fabric surfaces without wetting them. However, the bare fabrics were soaked up after the droplets spread on the surface. The initial contact angle measurements both sets of coated fabrics revealed values up to 150°, denoting superhydrophobicity.

**Fig. 5:**
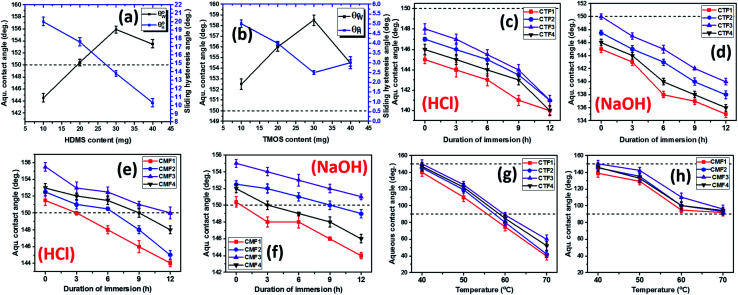
The wetting behaviors of cotton fabrics with varying HMDS (a) and TMOS (b) silylating coating contents without treatments. The effects of corrosive HCl (c, e) and NaOH (d, f) solvents and varying thermal conditions (g, h) on the surface wettability of coated superhydrophobic cotton fabrics.

Fig. 5 depicts the variation of aqueous contact 
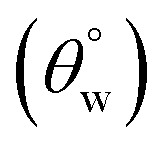
 and sliding hysteresis 
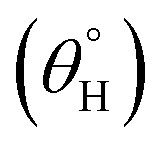
 angles of coated fabrics. The trend of results reveals consistent influence of silylated coating contents on surface wettability. Surface hydrophobicity increased with HMDS content (10–40 mg HMDS) while TMOS concentration kept unchanged (10 mg) within the coating. The coated fabrics in this study demonstrated significant superhydrophobicity at values of static 
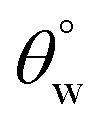
 more than 150°, except for CTF1 with 10 mg HMDS which also recorded the highest contact angle hysteresis (20° ± 0.5°). Fig. 5(a) reveals increase 
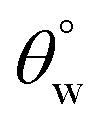
 values in the order: CTF3 (155.9° ± 0.6°) > CTF2 (150.4° ± 0.5°) > CTF1 (144.5° ± 0.7°) with 30, 20 and 10 mg HMDS, respectively. Other than CTF4 (153.5° ± 0.7°), increment in HMDS content within the coating significantly improved the superhydrophobicity of the fabric surface. CTF3 demonstrated superior protection with recorded magnitudes of values of 
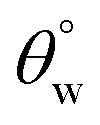
 and 
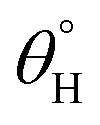
 up to 155.9° ± 0.6° and 13.8° ± 0.3°. With HMDS, the rough and porous nanostructures of the coated surface was enriched with stable substituted silyl (R_3_Si) groups. These features contributed to improved hydrophobicity, beyond which the surface fabric wettability increased, and this could have been due to saturation of the nanocomposite coating pores with HMDS. At this stage, these surface change had become unfavorable toward the formation trapped air within nanostructure of the coating, hence, disrupting Cassie–Baxter surface tension between the sitting liquid drop and the surrounding air.^2,4^

To investigate the effect of TMOS content on surface wettability, bare fabrics were modified with coatings containing 10–40 mg TMOS mixed with a fixed HMDS concentration (10 mg); labelled CMF1–4. The magnitudes of surface contact angle 
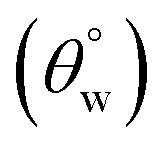
 were also measured. The trend of this parameter noticeably increased with TMOS content within the coated fabric up to 30 mg (being the optimum concentration of silylating agent offering the most superhydrophobicity). Compared to CMF3, the value of 
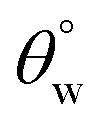
 for CMF4 was less due to overpacked coating network with silica/PDMS nanocomposite with stable non-hydrolysable octadecyl moieties. From Fig. 5(b), higher 
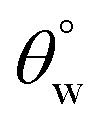
 values (158.5° ± 0.5°) were recorded for CMF3 with 30 mg TMOS while the least was recorded for 10 mg TMOS (152.5° ± 0.5°). The aqueous rolling angle was also observed to reduce with TMOS content; the least and most recorded magnitudes of 
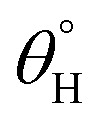
 were 2.5° ± 0.1° for CMF3 and 5.0° ± 0.2° for CMF1. CMF1–4 coated fabric exhibited superior superhydrophobicity compared to CTF1–4 since their surfaces still maintained inherent superhydrophobicity with values of 
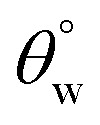
 greater than 150° as well as values of 
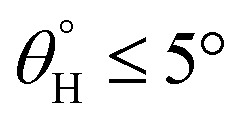
. The contribution of silylation to surface wetting mechanism for CMF1–4 and CTF1–4 coated fabrics is presented within the ESI (Fig. S2).†

### 3.4. Examining the chemical and thermal stabilities of silylated superhydrophobic coated fabric filters

Since the coated fabrics would be utilized is various service environments with several inherent factors capable of affecting their protective performances,^26^ stability tests were conducted in 1 M HCl and NaOH. These tests involved the complete immersion of the coated fabrics in 50 mL test solutions for 24 h. After each test within respective solvent, each fabric was repeatedly rinsed with absolute ethanol, dried in vacuum for 1 h at 35 °C before measuring its surface aqueous contact angles. Fig. 5(c–f) depicts the trend of 
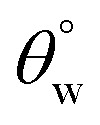
 values after exposure to HCl and NaOH solvents. In both corrosive media, CTF1–4 coated fabrics performed less than CMF1–4 as observed in the decreased magnitudes of 
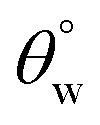
. Unlike CTF1–4, CMF1–4 coated fabrics still retained their superhydrophobicity to some extent, hence their superior protective properties, since their 
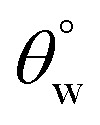
 values stood above 150°. The reason for this could be linked with influences of the long octadecyl C18 moiety from the trimethoxy(octadecyl)silane (TMOS) precursor that contributed to the stable superhydrophobic character of these coated fabric relative to HMDS modification.^27^ However, values of 
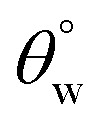
 significantly reduced less than 150° for coated fabrics with lesser TMOS content (CMF1–2 with 10 and 20 mg TMOS and 10 mg HMDS) between 0 and 12 h due to the corrosive impact of both solvents (HCl and NaOH). Though both solvents impacted on these coated fabrics, NaOH had the most effect relative to HCl. In both solvents, higher performances were recorded for coated fabrics with 30 mg TMOS/10 mg HMDS (CMF1) as well as 30 mg HMDS/10 mg TMOS (CTF3) due to their unique surface chemistry and contribution of their nanostructures. Normally, the polysiloxane networks within most coatings of this nature show significant chemical resistance.

In the test for thermal stability, all coated fabrics were exposed to defined thermal conditions in vacuum between 40 and 70 °C for an hour before measuring their magnitudes of 
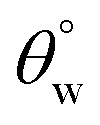
. The results of thermal stabilities of these silylating superhydrophobic coated fabrics are presented in Fig. 5(g and h). Between both coating systems, there was significant reduction in superhydrophobicity due to the impact heat. This must have led to loss of protective performance due to breakdown of polymer chains and loss of coating-fabric adhesion.^28,29^ However, CMF1–4 coated fabrics still exhibited hydrophobicity 
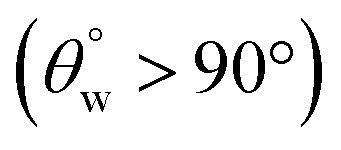
 compared to CTF1–4 due to increased silica content. The observed decrease in 
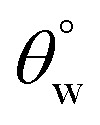
 could be linked with pyrolysis of their organic PDMS phase and it was more for CTF1–4 than CMF1–4. At 70 °C, there was complete degradation of these surface CTF1–4 coating, however, CTF4 still showed higher 
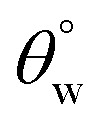
 magnitudes. Intense heat could have also contributed to loss of fabric bound hydroxyl chemical groups beneath these coated.^4^ Coatings with higher TMOS contents showed more surface stability due to the low thermal expansion and high thermal stability of the sol–gel derived polysiloxane coating network (*n*Si–O–Si). The incorporation of this secondary phase contributed to stable thermal shock resistant and conductivity unlike the organic HMDS presence that only increased superhydrophobicity.

### 3.5. Self-cleaning capacities of silylated superhydrophobic coated fabric filters

**Fig. 6:**
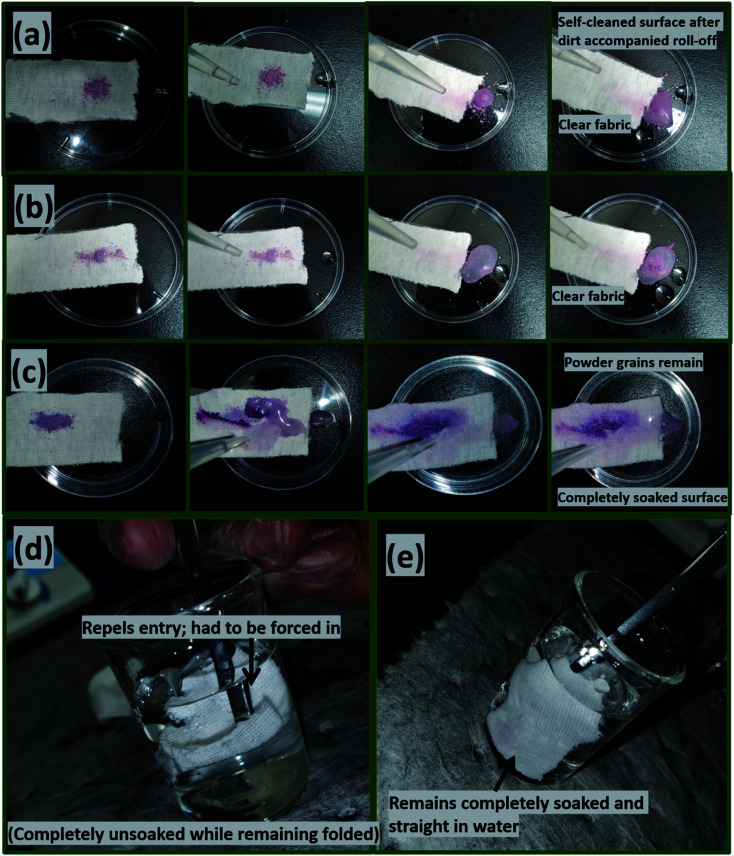
Optical images showing varying wetting behaviours of self-cleaning coated superhydrophobic fabrics (a, CTF3 and b, CMF3) compared to bare fabric (c). The wetting behaviours of coated superhydrophobic (d) and bare (e) fabrics during complete submersion in water.

In this section, the variants of coated fabrics with the best superhydrophobic performance were chosen; CTF3 (with 30 mg HMDS/10 mg TMOS) and CMF3 (with 30 mg TMOS/10 mg HMDS). To investigate the self-cleaning capacities of these coated fabrics, finely divided powdery grains of colored industrial chalks were placed on these coated fabrics. Fig. 6 depicts the optical images showing varying wetting behaviours of self-cleaning silylated superhydrophobic coated fabrics (a, CTF3 and b, CMF3) compared to bare fabric (c). These setups were then tilted at defined gradients on glass slides inserted in preclean Petri dishes before carefully introducing water droplets. Here, these droplets were allowed to slide through these powdery dust-leavened coated surfaces (a, b), in turn leaving clear paths of free of adhering dirt particles. The superhydrophobicity of these self-cleaned surfaces allowed for inherent removal of powdery debris. Conversely, Fig. 6(c) depicts significant surface wetness on the bare fabric after water adsorption. This hydrophilic character was due to the presence of cellulosic chemical groups on the fabric that also allowed for the adhesion of powdery dirt on the surfaces of this fabric without accompanied roll-off compared to the coated superhydrophobic fabrics. The reduced surface energies and higher hydrophobicity of coated surfaces contributed to the observed self-cleaning characters. This trend of results are direct contributions of incorporated silylated PDMS groups within these coated fabric surfaces. The whole fabric areas of both bare and coated fabrics showing their comparative wetting behaviours are depicted in Fig. 6(d). With the coatings, these non-wetted superhydrophobic fabrics quickly folded when forced into water and remained unsoaked due to creation of surface tension. This bright superhydrophobic coated surface remained unwetted in water as air is trapped within the roughened nano- and microporous fabric surfaces in water, in turn, leading to the reduced surface wetting.^4^ However, the bare fabric (Fig. 6(e)) rapidly adsorbed water, swelled and soaked up completely while it also remained straighten upon sinking unassisted into the bottom of the beaker. The observed surface property is consistent with superhydrophilicity when exposed to compared to coated fabrics under similar conditions.

### 3.6. Using silylated superhydrophobic coated fabric filters as oil–water separation filters

**Fig. 7:**
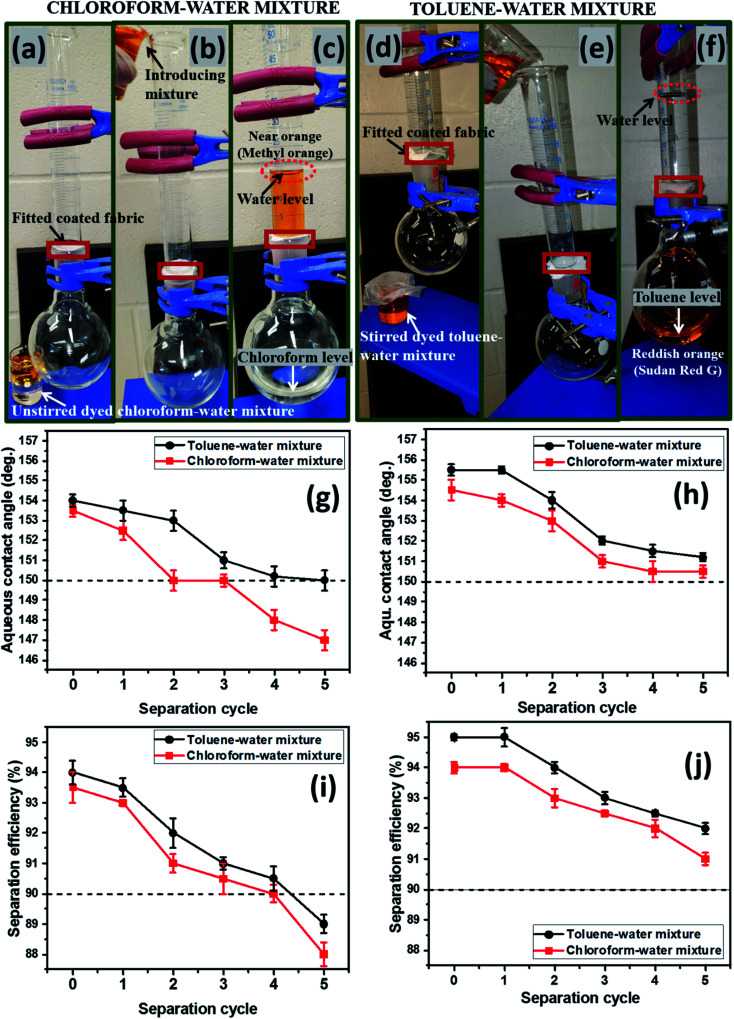
Laboratory fabricated oil–water filtration setups showing (a, d) fitted silylated superhydrophobic CMF3 coated fabric alone; (b, e) introduction of the heavy oil–water and light oil–water mixtures prior to the filtration process; (c, f) completely separated oil and water after several filtration cycles. (g, h) Aqueous contact angle fluctuations and (i, j) separation efficiency for the best coated superhydrophobic fabrics (left: CTF3 with 30 mg HMDS/10 mg TMOS and right: CMF3 with 30 mg TMOS/10 mg HMDS) between 0 to 5 separation cycles.

As presented in Fig. 7(a and d), the filtration setup fitted silylated superhydrophobic coated fabrics was utilized in oil–water separation. As modeled oil samples, chloroform (heavy oil) and toluene (light oil) were chosen due to their difference in densities when mixed with water. After introduction of both heavy oil–water and light oil–water mixtures prior to the filtration process (b, e), separation of phases occurred according to the filtration cycle. In the case of chloroform, it separated and sank readily to the round-bottom flask as a colorless liquid, leaving the dyed aqueous phase on the upper glass over the coated silylated superhydrophobic fabric after several repeated filtration cycles (c, f). A similar test was also conducted for dyed toluene and this latter organic solvent also remained immiscible in water but less dense, so it floated above the aqueous layer. Summaries of the aqueous contact angle fluctuations (g, h) of the best performed coated superhydrophobic fabrics (CTF3 with 30 mg HMDS/10 mg TMOS and CMF3 with 30 mg TMOS/10 mg HMDS) are depicted in Fig. 7. These results were collected after exposure to chloroform and toluene at defined separation cycles. In this study, chloroform was observed to be heavier than toluene while the water is denser than toluene. For both organic solvents, the coated cotton fabrics for this test retained their superhydrophobicity 
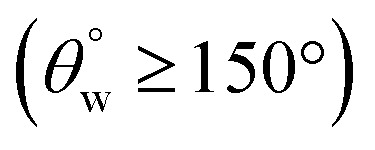
 even after separation cycles under study, except for CTF3 (with 30 mg HMDS/10 mg TMOS) after the fourth and fifth consecutive separation cycles in chloroform–water mixture. The least recorded values of 
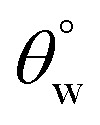
 were 150.0° ± 1.5° and 147.1° ± 0.5° after the fifth filtration cycles in toluene and chloroform, respectively, for CTF3 while 151.1° ± 1.5° and 150.5° ± 1° were recorded for CMF3 at the same cycle. For maintaining contact angles up to 150°, these superhydrophobic coated fabrics demonstrated significant qualities for reusability and recyclability during filtration. Between both organic solvents, the surfaces of fabrics treated with chloroform were most impacted by the solvent treatments compared to toluene within the separation cycles under study. CMF3 remained the coated fabric with superior performance compared to CTF3 due to the formation of stable non-hydrolysable octadecyl moiety on nanosilica phases within the PDMS matrix at optimum trimethoxy(octadecyl)silane content. This alkyl moiety from the TMOS also provides the required low surface energy to the coating matrix needed to retain its superhydrophobicity. The computed values of chloroform–water and toluene–water separation efficiencies are presented in Fig. 7(i and j) between 0 and 5 cycles. In all, both silylated superhydrophobic coated fabrics retained separation efficiencies over 90%. CMF3 recorded separation efficiencies of 94 and 95% for chloroform and toluene, respectively, while those of CTF3 stood at 88 and 89%, in that order. The observed reduction in separation efficiency for both coated fabrics may be linked with the complete saturation of inherent nanopores after numerous cycles. This was possible since this coated fabric acted as a filter membrane with opposite wettability to aqueous phase while allowing for preferential absorption of the organic oil phase.^4,30,31^ The observed degree of permeation of solvents through the nanopores of these coated fabrics in this test was due to differences in solvent weights. The observed trend could also be attributed to gravity-assisted percolation as well as the contributions of superhydrophobic coated fabrics.^4^ Comparative barrier performances of some recent PDMS-based coated cotton fabrics within this study and in those reported the literature is presented in Table 3.^32–39^

**Table 3:** Comparative barrier performances of some PDMS-based superhydrophobic coated cotton fabrics in this study and those within the literature

S/no.	Type of base film system (coating additive)	Coating technique	Superhydrophobic?^a^	Capacity for self-cleaning?	Capacity for oil–water separation?^b^	Coating performance is attributed to the following reason(s)	Ref.
1.	PDMS/siloxane coating (hexamethyldisilazane and trimethoxy(octadecyl)silane silylating agents)	Dip coating method	Yes 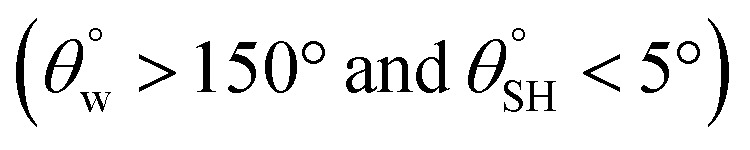	Yes	Yes (>90%)	Superhydrophobic coating surfaces modified with silylating chemical groups leavened with nanosilica reinforced morphologies and low surface energy polysiloxane chemical groups on the cotton fabrics	This study
2.	PDMS/polyvinyl alcohol (SiO_2_NPs)	Dip dry coating method	Yes 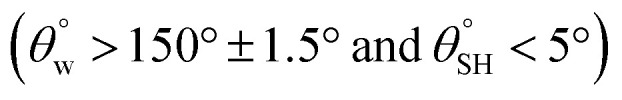	Yes	Yes (>95%)	Silica/PDMS modification on the textile surface created a lower surface energy than unmodified textile	4
3.	PDMS based coating (SiO_2_NPs)	Dip coating method	Yes 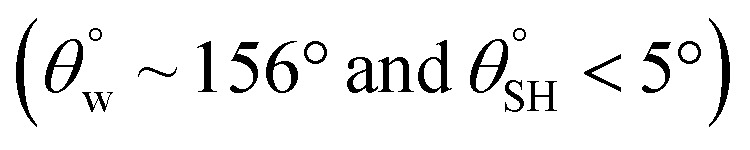	Yes	Yes (>95%)	Formation of cross-linked PDMS network with longer polymer chains interlocked with nanoparticles, forming a robust superhydrophobic coating surface	13
4.	Alkylammonium functional silsesquioxane/PDMS (TiO_2_NPs)	Spraying method	Yes 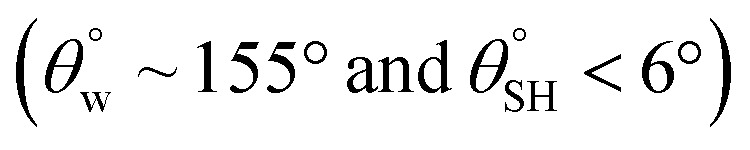	Yes	Yes (>99%)	Superhydrophobic PDMS/TiO_2_ hybrid coating surface with low surface energy	14
5.	Octa vinyl polyhedral oligomeric silsesquioxane/PDMS nanocomposite coating system (TiO_2_NPs)	Spraying method	Yes 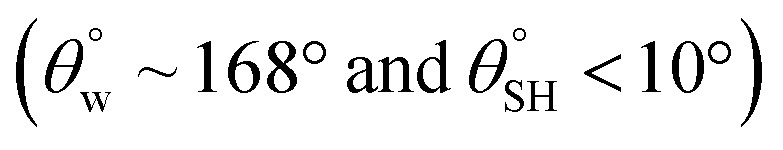	Yes	Not measured	Roughness enhancement from TiO_2_ clusters and OV-POSS structures; reduced surface energy related to low surface tension of vinyl groups and PDMS content	32
6.	PDMS based coating (AgNWs)	Dip coating method	Yes ( 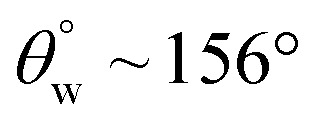 ; the values of 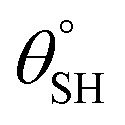 was not measured)	Yes	Yes (95.6%)	The functionalized AgNWs with PDMS groups offered superhydrophobic and lower surface energy on coated cotton fabric	33
7.	PDMS/polyimide (AgNPs)	*In situ* dip coating method	Yes ( 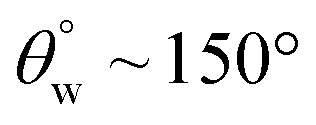 ; the values of 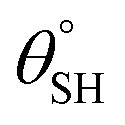 was not measured)	Yes	Not studied	The introduction of polydopamine increased fabric-AgNP bonding, thereby preventing silver nanoparticles from falling off the surface, hence increasing superhydrophobicity in the presence of PDMS	34
8.	PDMS/silica (nano-silica)	*In situ* sol–gel coating method	Yes ( 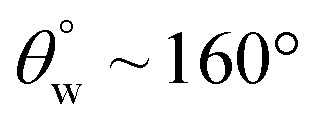 ; the values of 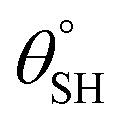 was not measured)	Yes	Not studied	Incorporation of nano-silica within superhydrophobic PDMS coating contributed to lowered surface energy	35
9.	PDMS based coating (MWCNTs)	*In situ* dip coating method	Yes ( 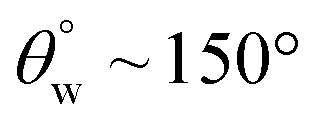 ; the values of 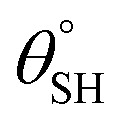 was not measured)	Yes	Yes (>94%)	Incorporation of MWCNTs within PDMS coating contributed to lowered surface energy and superhydrophobicity	36
10.	PDMS based coating (ZnO)	Dip coating method	Yes ( 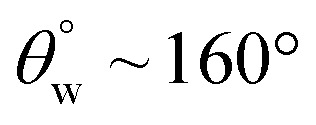 ; the values of 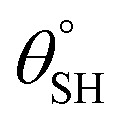 was not measured)	Yes	Yes (>95%)	Incorporation of ZnO within PDMS coating contributed to lowered surface energy and superhydrophobicity	37

a Highest and lowest recorded 
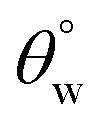
 and 
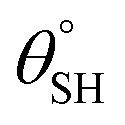
, respectively.

b Highest recorded separation efficiency.

## 4. Conclusions

New superhydrophobic self-cleaning siloxane/polydimethylsiloxane hybrid nanocomposite coatings were synthesized using two distinct silylating agents (HMDS and TMOS) to achieve enhanced superhydrophobicity when deposited on cotton fabrics. Variants of these coatings were also prepared by varying the amounts of both silylating coating components. After characterization and testing, the following conclusions were derived from the results of the experiments:

1. The hydrophilic cellulosic chemical groups on the uncoated cotton fabric contributed to its ability to readily adsorbed water. However, the coated silylated superhydrophobic coated fabrics demonstrated unique self-cleaning capacities due to their low surface energies and highly reduced surface wettability.

2. Coated cotton fabrics with higher TMOS contents (CMF1–4) demonstrated significant resistance against thermal oxidation due to their increased nanosilica composition. However, there was also reduced magnitudes of 
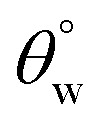
 at higher treatment temperatures. These coated fabrics were still hydrophobic 
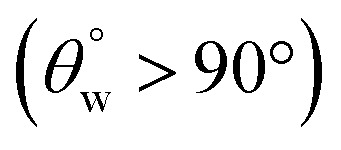
 at the end of the tests. However, for CTF1–4 matrix (with higher HMDS content), the aqueous contact angle reduced remarkably after treatment to temperatures up to 40–70 °C due to pyrolysis of their organic contents.

3. Coated fabrics with higher TMOS content did not retain their superhydrophobicity 
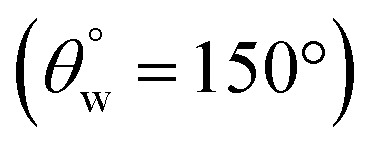
 after acid and alkaline treatments. Both corrosive solvents had detrimental effects on both sets of coated fabrics within the duration of the test.

4. The observed superhydrophobic characters, solvent and thermal stabilities, capacities for self-cleaning and oil–water separation are ascribed to their nanopatterned nanosilica coated morphologies and low surface energy silylated hybrid polysiloxane chemical groups on these coatings.

5. Coated superhydrophobic fabrics with 30 mg TMOS/10 mg HMDS (CMF3) and 30 mg HMDS/10 mg TMOS (CTF3) exhibited the optimal superhydrophobicity. Both fabrics also retained percentage separation efficiencies over 90% for both chloroform–water and toluene–water filtered mixtures. However, CTF3 displayed less than 
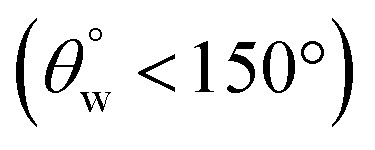
 with recorded separation efficiency less than 90° at the fifth consecutive separation cycle.

6. The substituted silyl (Me_3_Si) and octadecyl chemical groups on HMDS and TMOS contributed to enhanced superhydrophobicity. However, it was the hydrolysable trimethoxy silyl ((MeO)_3_Si) chemical groups on TMOS that allowed for the formation of nanosilica with Si–O–Si linkages needed to foster stable coatings.

## Symbols, abbreviations and acronyms

*γ*_LA_
: Surface energy of liquid–air interface
*γ*_SA_
: Surface energy of the surface–air interface
*γ*_SL_
: Surface energy of surface–liquid interface
*θ*_o_
: Contact angle of water on the surface
*R*_f_
: Ratio of surface area of rough surface to the surface area of a flat projection of the same surface
*f*_LA_
: Liquid–air fraction, the fraction of the liquid droplet that is in contact with air
*θ*_CB_
: Contact angle of water predicted by Cassie–Baxter's model
*θ*
: Contact angle of water predicted by Wenzel's model
AgNPs: Silver nanoparticles
AgNWs: Silver nanowires
EPMM: 3-(2,3-Epoxypropoxypropyl)methyldimethoxysilane
EtPDMS: Ethoxy terminated polydimethylsiloxane
FTIR: Fourier transform infrared
HMDS: Hexamethyldisilazane
MWCNTs: Multiwalled carbon nanotubes
NPs: Nanoparticles
SiO_2_NPs: Silicon oxide nanoparticles
TiO_2_NPs: Titanium oxide nanoparticles
TMCS: Chlorotrimethylsilane
TMOS: Trimethoxy(octadecyl)silane
UHV: Ultra-high vacuum
XPS: X-ray photoelectron spectroscopy
ZnO: Zinc oxide

## Conflicts of interest

There are no conflicts of interest.

## Supplementary Material

RA-011-D0RA10565A-s001
