# Supplementary material for: Effect of silylating agents on the superhydrophobic and self-cleaning properties of siloxane/polydimethylsiloxane nanocomposite coatings on cellulosic fabric filters for oil–water separation
Source: RSC Adv. 2021 Mar 4;11(16):9586–99. doi: 10.1039/d0ra10565a (PMC8695392; doi:10.1039/d0ra10565a)
Supplement: RA-011-D0RA10565A-s001 [file RA-011-D0RA10565A-s001.pdf]

Electronic Supplementary Material (ESI) for RSC Advances.
This journal is © The Royal Society of Chemistry 2021

Effect of silylating agents on the superhydrophobic and self-cleaning characters of siloxane/polydimethylsiloxane nanocomposite coatings on cellulosic fabric filters for oil-water separation

Ubong Eduok*[1], Omar Faye[1], Jerzy Szpunar[1], Mazen Khaled[2]

1. *Department of Mechanical Engineering, College of Engineering, University of Saskatchewan, 57 Campus Drive, Saskatoon, S7N 5A9, Saskatchewan, Canada.*
2. *Department of Chemistry and Earth Sciences, College of Arts and Sciences, Qatar University, P.O. Box 2713, Doha, Qatar*

SUPPORTING INFORMATION

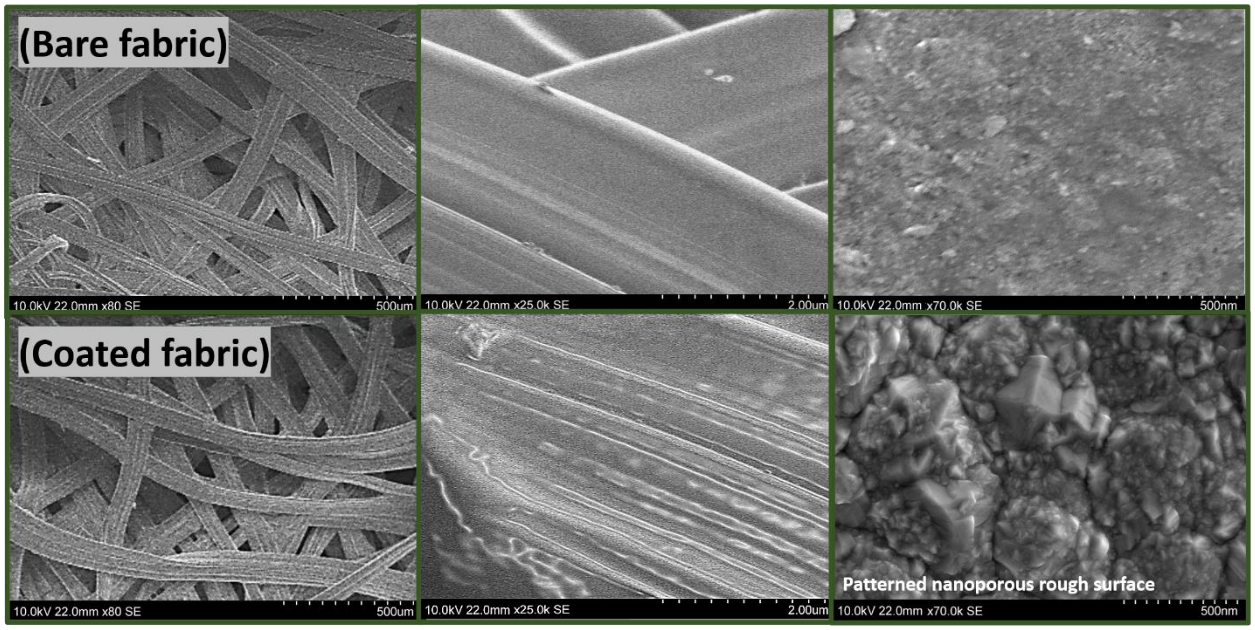

Fig S1. Low-and high-magnification FESEM micrographs of bare and coated (CMF3) superhydrophobic fabrics.

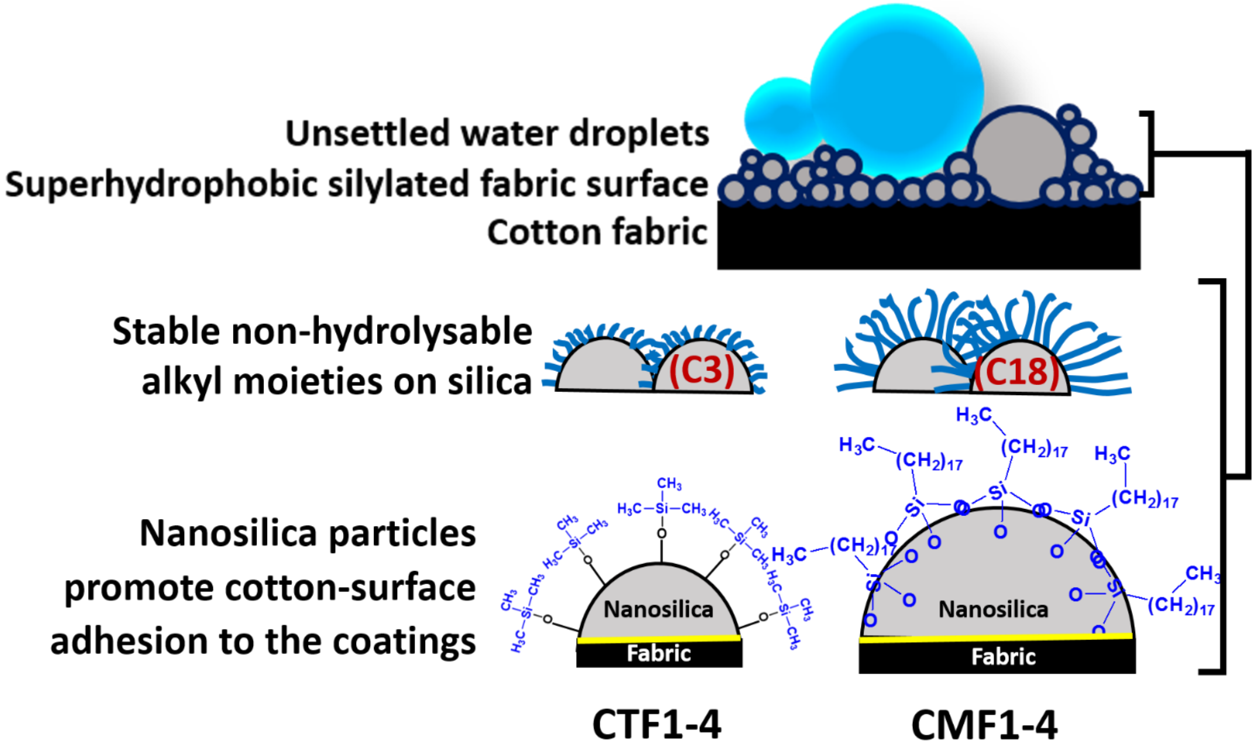

Fig S2. The contribution of silylation to surface wetting mechanism for CMF1-4 and CTF1-4: CMF1-4 coated fabrics demonstrated superior performance compared to CTF1-4 due to the formation of stable non-hydrolysable octadecyl moiety on the nanosilica phases within the PDMS matrix. This is due to the presence of trimethoxy(octadecyl)silane (TMOS). This alkyl moiety from TMOS also provided the required low surface energy to the coating matrix needed to retain its superhydrophobicity compared to HDMS in CTF1-4. Unlike HMDS with substituted silyl (Me_3Si) groups, TMOS consists of hydrolysable trimethoxy (MeO_3Si) chemical groups that allowed for the formation of nanosilica with Si–O–Si linkages needed to foster stable coatings. These nanosilica particles also promoted cotton-surface adhesion to the coating via Si–O–Si linkages (see yellow lines).
